# Supercontinuum generation in a nonlinear ultra-silicon-rich nitride waveguide

**DOI:** 10.1038/s41598-022-13734-9

**Published:** 2022-06-08

**Authors:** Yanmei Cao, Byoung-Uk Sohn, Hongwei Gao, Peng Xing, George F. R. Chen, Doris K. T. Ng, Dawn T. H. Tan

**Affiliations:** 1 Photonics Devices and Systems Group, Singapore University of Technology and Design, 8 Somapah Road, Singapore, 487372 Singapore; 2 Institute of Microelectronics, A*STAR, 2 Fusionopolis Way, #08-02, Innovis Tower,, Singapore, 138634 Singapore

**Keywords:** Nonlinear optics, Integrated optics, Supercontinuum generation

## Abstract

Supercontinuum generation is demonstrated in a 3-mm-long ultra-silicon-rich nitride (USRN) waveguide by launching 500 fs pulses centered at 1555 nm with a pulse energy of 17 pJ. The generated supercontinuum is experimentally characterized to possess a high spectral coherence, with an average |*g*_12_| exceeding 0.90 across the wavelength range of the coherence measurement (1260 nm to 1700 nm). Numerical simulations further indicate a high coherence over the full spectrum. The experimentally measured supercontinuum agrees well with the theoretical simulations based on the generalized nonlinear Schrödinger equation. The generated broadband spectra using 500 fs pulses possessing high spectral coherence provide a promising route for CMOS-compatible light sources for self-referencing applications, metrology, and imaging.

## Introduction

Supercontinuum is an ultrabroad band spectrum that has significant applications in many fields, including optical communications^1^, high-precision frequency metrology^2^, and optical coherence tomography^3^. It’s typically generated by injecting ultrashort and intense pulses into a nonlinear material where they experience an extreme spectral broadening caused by nonlinear mechanisms, for instance, self-phase modulation (SPM), high-order soliton fission, and dispersive wave generation.

Supercontinuum generation (SCG) has been extensively investigated and made substantial progress in many on-chip platforms, including silica^4,5^, Si^6–9^, amorphous Si^10,11^, lithium niobate^12^, AlGaAs ^13^ and SiN_x_^14–17^. Amongst these, silicon was studied intensively as a promising platform because of its CMOS-compatibility, high Kerr nonlinearity (6 × 10^–14^ cm^2^ W^–1^), and large refractive index contrast with SiO_2_ cladding which enables strong mode confinement. However, the small bandgap of silicon (1.12 eV) leads to two-photon absorption (TPA) at telecommunication wavelengths. Silicon nitride (Si_3_N_4_) is another widely studied platform for supercontinuum generation, which has negligible TPA in the near-IR, but its low Kerr nonlinearity (2.5 × 10^–15^ cm^2^ W^–1^) limits the extent of spectral broadening at low pulse energies.

USRN (Si_7_N_3_) is backend CMOS-compatible, with a linear refractive index of 3.1 and a Kerr nonlinearity of 2.8 × 10^–13^ cm^2^ W^–1^^18^. Owing to its high silicon content, the Kerr nonlinearity of USRN is 100 times larger than that of silicon nitride ^14,16^ and silicon-rich nitride ^17^. In addition, USRN has a large bandgap (2.1 eV) allowing for nonlinear interactions free from TPA at telecommunication wavelengths^19–21^. All these benefits make USRN an ideal platform for on-chip SCG.

SCG has been previously demonstrated in USRN devices^18,22–24^. For example, a 620 nm-spanning supercontinuum at the –30 dB level was observed using 500 fs pulses in a 7-mm-long USRN waveguide^18^. A spectral broadening with a 30 dB bandwidth of 340 nm was also demonstrated using 1.7 ps pulses in a two-stage USRN Bragg grating device, which consists of a 1-mm-long Bragg grating used to trigger Bragg soliton-effect compression and fission, followed by a 6-mm-long USRN channel waveguide where enhanced spectral broadening occurs^24^. Importantly, that work featured solitons of low soliton order, facilitating the attainment of high spectral coherence.

In this work, we experimentally and numerically demonstrated a SCG with high coherence in a USRN waveguide. Compared with many other SCG demonstrations, this work relaxes the pulse width requirement from tens of femtoseconds to 500 fs, while requiring one of the lowest pulse energies.

## Results

**Figure 1:**
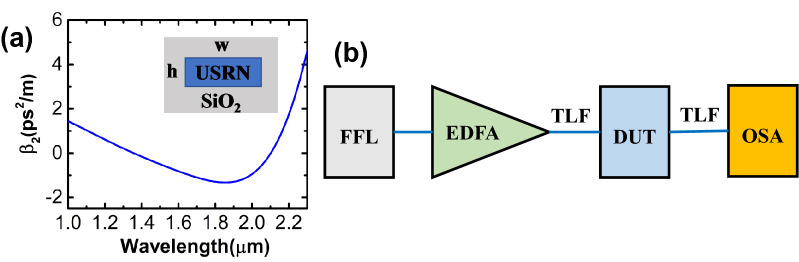
(**a**) Calculated group velocity dispersion (GVD) curve for the fundamental TE mode of a USRN channel waveguide. The insert shows the cross section of the USRN waveguide with a width *w* = 600 nm and a height *h* = 400 nm. (**b**) Schematic of the experimental setup for supercontinuum generation in the USRN waveguide, where blue lines denote the polarization maintaining fibers, where FFL: femtosecond fiber laser, EDFA: erbium-doped fiber amplifier, DUT: device under test, TLF: tapered lensed fiber, OSA: optical spectrum analyzer.

Waveguide dispersion is essential to SCG at a specific input pulse wavelength, which can be modulated by tailoring the width and height of waveguides. Based on the 1555 nm pulse wavelength, our USRN waveguide is designed to have a wide anomalous group-velocity dispersion (GVD) region near 1555 nm. Figure 1a shows the calculated GVD as a function of wavelength for the fundamental TE mode of a USRN channel waveguide, which is 600 nm wide and 400 nm high. As shown in Fig. 1a, anomalous dispersion occurs near 1555 nm, conducive to a broadband spectrum caused by self-phase modulation (SPM), soliton fission, and dispersive wave generation.

The schematic of the experimental setup for supercontinuum generation in the USRN waveguide is shown in Fig. 1b. In the experiment, a mode-locked femtosecond fiber laser is utilized to launch 500 fs pulses with a repetition rate of 20 MHz centred at 1555 nm, followed by an erbium-doped fiber amplifier (EDFA). The pulses are adjusted for quasi-TE polarization, and coupled into the 3-mm long USRN waveguide with tapered lensed fibers. The coupling losses are estimated to be 6–7 dB per facet, and the propagation loss is around 3 dB/cm extracted from cutback measurements. The output spectra from the waveguide are separately monitored utilizing two different optical spectrum analyzers (OSAs), one for the wavelength range from 800 to 1750 nm, and the other for the wavelength range from 1600 to 2400 nm.

**Figure 2:**
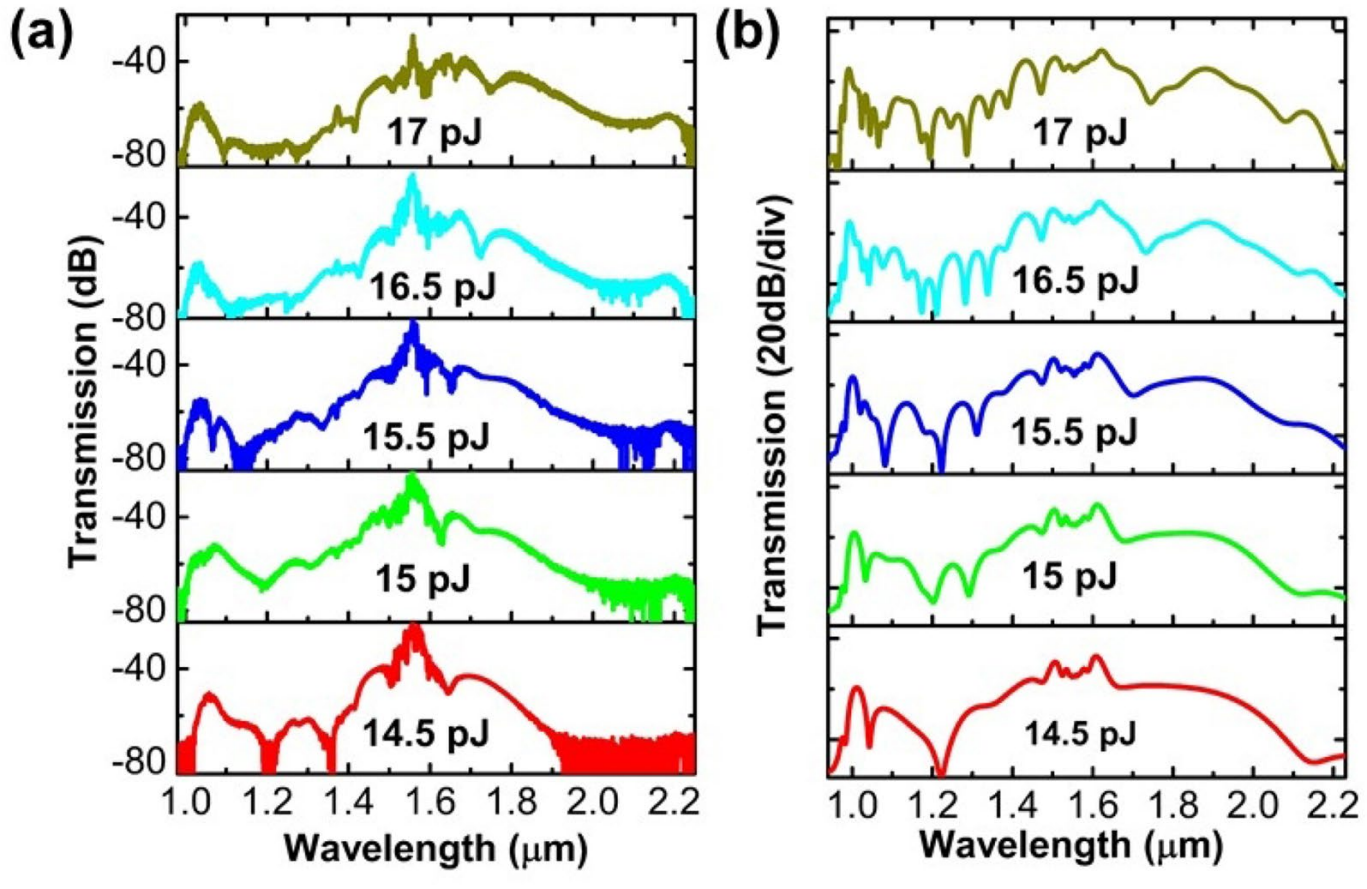
Measured (**a**) and simulated (**b**) output spectra at various pulse energies coupled into the USRN waveguide.

**Figure 3:**
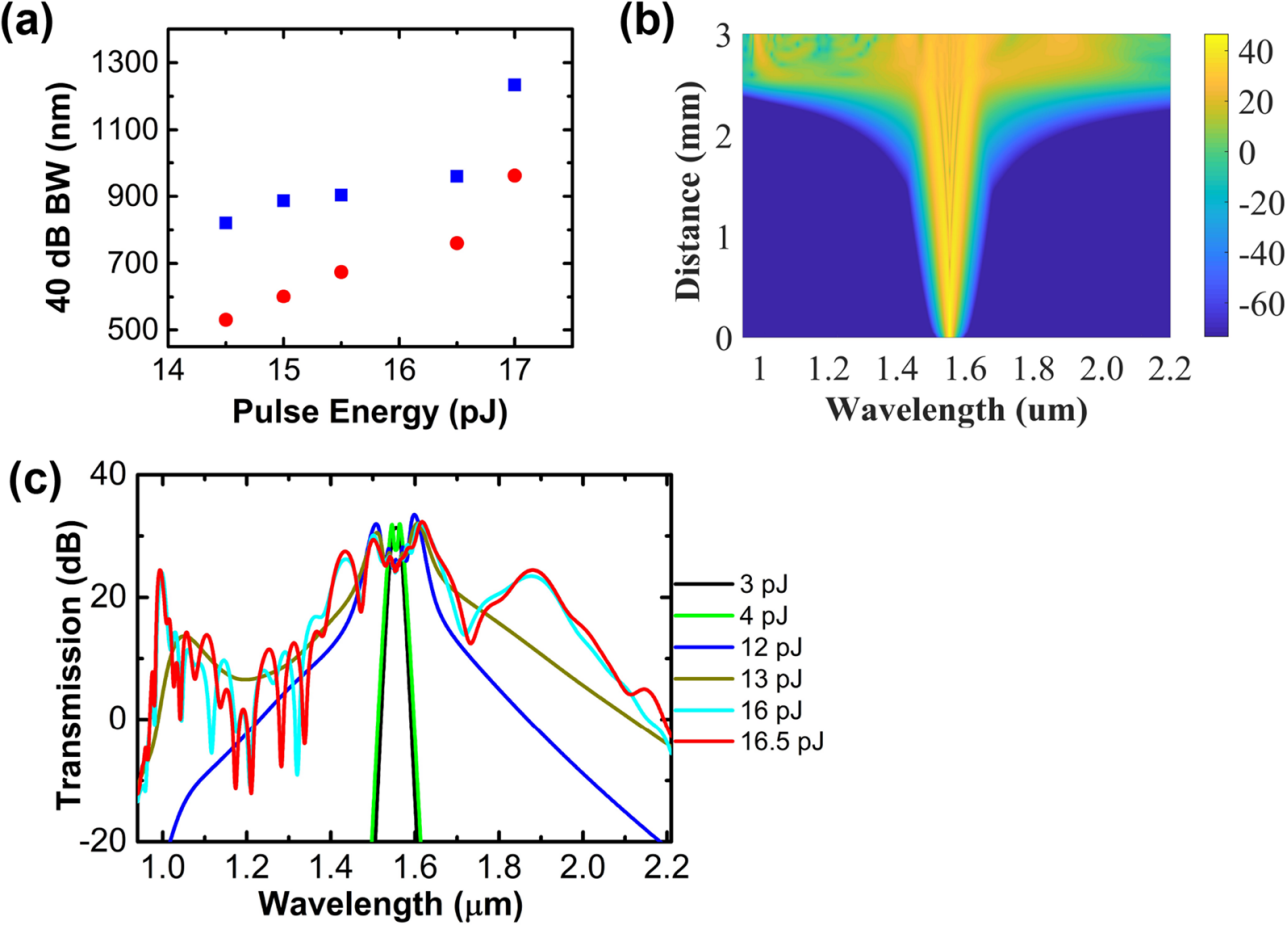
(**a**) Experimentally measured 40 dB bandwidth at various input pulse energies, where the blue data are obtained based on the SCG span within the –40 dB level edges, while the red data are obtained based on the SCG span after subtracting components below the –40 dB level. (**b**) Simulated pulse dynamics using 500 fs pulses in the USRN waveguide. (**c**) Simulated output spectra at various pulse energies coupled into the USRN waveguide.

Figure 2a shows the measured output spectra at various pulse energies coupled into the USRN waveguide. As can be seen in Fig. 2a, the spectral broadening increases with the input pulse energy, and dispersive waves can be seen at both the short wavelength side (~ 1000 nm) and long wavelength side (~ 2200 nm) at higher pulse energies. These dispersive waves are important for extending the broadness of the SCG and originate from energy transfer from solitons generated during the process to the dispersive wave^25–28^. At the maximum pulse energy of 17 pJ, a supercontinuum with a lower edge of 1006 nm and an upper edge of 2240 nm at the –40 dB level is obtained, although some of the spectral components ranging from 1072 to 1344 nm are below this level. At the –20 dB and –30 dB levels, the generated supercontinuum has a bandwidth of 392 nm (1470 nm to 1862 nm) and 530 nm (1427 nm to 1957 nm), respectively. The experimentally measured 40 dB bandwidth at various pulse energies is plotted in Fig. 3a, where the blue data are obtained based on the SCG span within the –40 dB level edges, while the red data are obtained based on the SCG span after subtracting components below the –40 dB level. It can be seen in Fig. 3a that the 40 dB bandwidth increases with the pulse energies coupled into the USRN waveguide.

To better understand the pulse evolution dynamics in the USRN waveguide, we performed the numerical simulations using the Generalized Nonlinear Schrödinger Equation (GNLSE), as shown in Eq. (1) ^29^:

1 $$\frac{\partial A}{{\partial z}} = \sum\nolimits_{k = 2}^{7} {\frac{{i^{k + 1} }}{k!}\beta_{k} \frac{{\partial^{k} A}}{{\partial t^{k} }}} - \frac{\alpha }{2}A + i\gamma (1 + i\tau_{shock} \frac{\partial }{\partial t})\left( {\left| A \right|^{2} A} \right)$$

This equation is solved using the split-step Fourier method. In Eq. (1), *A* (*z*, *t*) is the slowly varying pulse envelope, a good approximation in this regime. *A* (0, *t*), the initial input pulse, is assumed to be a hyperbolic-secant field, which has a peak power *P* = 34 W (corresponding to a pulse energy of 17 pJ used in the experiment), a temporal pulse width of 500 fs, and a center wavelength of 1555 nm. We used the same input pulse parameters in the simulations as that used in the experiment.

In the simulations, we take dispersion, linear loss, self-phase modulation (SPM), and self-steepening effects into consideration. As shown on the right side of Eq. (1), the first term accounts for the dispersion effects in the USRN waveguide, where *β*_*k*_ is the *k*th-order dispersion coefficient obtained by performing Taylor-series expansion of the propagation constant *β*(*ω*), where *β*(*ω*) = *n*_eff_(*ω*)*ω*/*c* and *n*_eff_(*ω*) is obtained with finite element algorithm. We used up to 7th order dispersion in the simulations, where *β*_*2*_ = –0.64 ps^2^/m, *β*_*3*_ = 2.4 × 10^–3^ ps^3^/m, *β*_*4*_ = –1 × 10^–6^ ps^4^/m, *β*_*5*_ = 1 × 10^–7^ ps^5^/m, *β*_*6*_ = –6.8 × 10^–10^ ps^6^/m, and *β*_*7*_ = 4.8 × 10^–12^ ps^7^/m. The second term shows the effect of linear loss, where *α* is 3 dB/cm, a value obtained experimentally. The nonlinear parameter, *γ* = (*ω*_0_·*n*_2_)/(*c*·*A*_eff_), where *n*_2_ is the nonlinear refractive index, *c* is the light speed in vacuum and *A*_eff_ is the effective mode area. The third term represents nonlinear effects, including the SPM effect and self-steepening effect, where *τ*_shock_ = 1/*ω*_0_ is the shock coefficient.

Figure 2b shows the simulated output spectra at various input pulse energies. As can be seen in Fig. 2b, the spectral broadening increases with the input pulse energies. At low pulse energies, the nonlinear effects in the USRN waveguide get stronger with the increase in pulse energy, and the spectral broadening is mainly caused by self-phase modulation (SPM). When the pulse energy is further increased, greater spectral broadening occurs from soliton fission and dispersive wave generation. The simulated output spectra in Fig. 2b show similar broadening trends and dispersive wave positions to the experimental results shown in Fig. 2a. Figure 3b shows the simulated pulse dynamics using 500 fs pulses in the 3-mm-long USRN waveguide. As shown in Fig. 3b, the spectrum broadens symmetrically at the very beginning (less than 2 mm) due to SPM dominating the pulse dynamics. As the pulse propagates further along the waveguide, greater broadening occurs from soliton fission and dispersive wave generation.

The dispersive waves generated at ~ 1000 nm and ~ 2200 nm play a role in the broadening of the SCG spectrum. To further elucidate the dynamics surrounding the onset and growth of the dispersive waves, we simulate the output spectra at different pulse energies coupled into the USRN waveguide. This is shown in Fig. 3c. It may be observed that at low pulse energy (less than 4 pJ), the spectrum broadens mainly due to the SPM effect. With increasing pulse energy, greater spectral broadening occurs from soliton fission and dispersive wave generation. As can be seen in Fig. 3c, the onset of soliton formation occurs when the pulse energy is larger than 12 pJ, where the spectrum broadens asymmetrically with pedestals characteristic of solitons forming. The dispersive wave at the short wavelength side (~ 1000 nm) begins to be significant when the pulse energy exceeds 13 pJ, while the dispersive wave at the long wavelength side (~ 2200 nm) begins to be significant when pulse energy exceeds 16.5 pJ. At this pulse energy, the dispersive waves on both the short and long wavelength side grow in amplitude beyond the –30 dB level, leading to considerable growth in SCG bandwidth. At 17 pJ, a soliton order of *N* = 43 is achieved, where $N = \sqrt {L_{d} /L_{nl} }$, $L_{d}$ is the dispersion length obtained by $L_{d} = T_{0}^{2} /\beta_{2}$, and $L_{nl}$ is the nonlinear length obtained by $L_{nl} = 1/\gamma P$^8^. Here, $T_{{0{ }}}$ is the temporal pulse width, $\beta_{2}$ is the group velocity dispersion at 1555 nm, $\gamma$ is the nonlinear parameter, and *P* is the peak power coupled into the waveguide. By calculation, $L_{nl} = 1/\gamma P = { }$ 0.067 mm, $L_{d} = T_{0}^{2} /\beta_{2}$ = 125.7 mm, and *N* $=$ 43. The soliton fission length at a pulse energy of 17 pJ can be further obtained using $L_{fission} = L_{d} /N$ = 2.9 mm, shorter than the SPM-induced modulation instability length, which is 4.1 mm calculated using $L \approx \sqrt {2L_{D} L_{nl} }$. The shorter soliton fission length indicates that soliton fission plays a more dominant role in the spectral growth over modulation instability. In addition, the modulation instability length exceeds the length of the waveguide (3 mm), so it may further be inferred that modulation instability does not play a strong role in the spectral broadening process.

**Figure 4:**
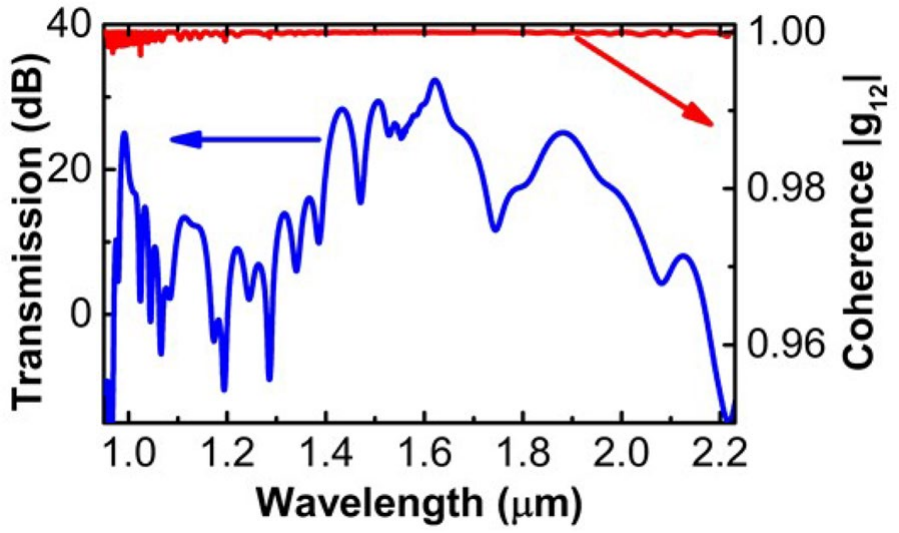
Simulated SCG spectrum (blue) and spectral coherence |*g*_12_| (red) by adding one-photon-per-mode noise using 500 fs pulses with a pulse energy of 17 pJ.

The coherence of the output spectra from the USRN waveguide is further studied both numerically and experimentally. In the numerical study, we simulated the pulse dynamics in the USRN waveguide using Eq. (1), by adding one photon per mode (OPM) quantum noise into the input pulse. We did 100 times simulation in total, and divided them into 50 pairs, where each pair has two different simulated spectra, the same method as reported in Ref.^26,30,31^. The spectral coherence *g*_12_(*λ*) may be calculated using

2 $$g_{12} (\lambda ) = {{E_{1}^{*} (\lambda )E_{2} (\lambda )} \mathord{\left/ {\vphantom {{E_{1}^{*} (\lambda )E_{2} (\lambda )} {\sqrt {\left| {E_{1} (\lambda )} \right|^{2} \left| {E_{2} (\lambda )} \right|^{2} } }}} \right. \kern-\nulldelimiterspace} {\sqrt {\left| {E_{1} (\lambda )} \right|^{2} \left| {E_{2} (\lambda )} \right|^{2} } }}$$

where *E*_1,2_ denote the individually simulated electric field, which is an averaged ensemble of the 50-pair simulations. Figure 4 shows the simulated SCG spectrum (blue line) and coherence |*g*_12_| (red line) by adding OPM noise using 500 fs pulses with a pulse energy of 17 pJ. From Fig. 4, the calculated coherence |*g*_12_| is close to 1 across most of the simulated wavelength range, indicating a high spectral coherence of the generated supercontinuum.

**Figure 5:**
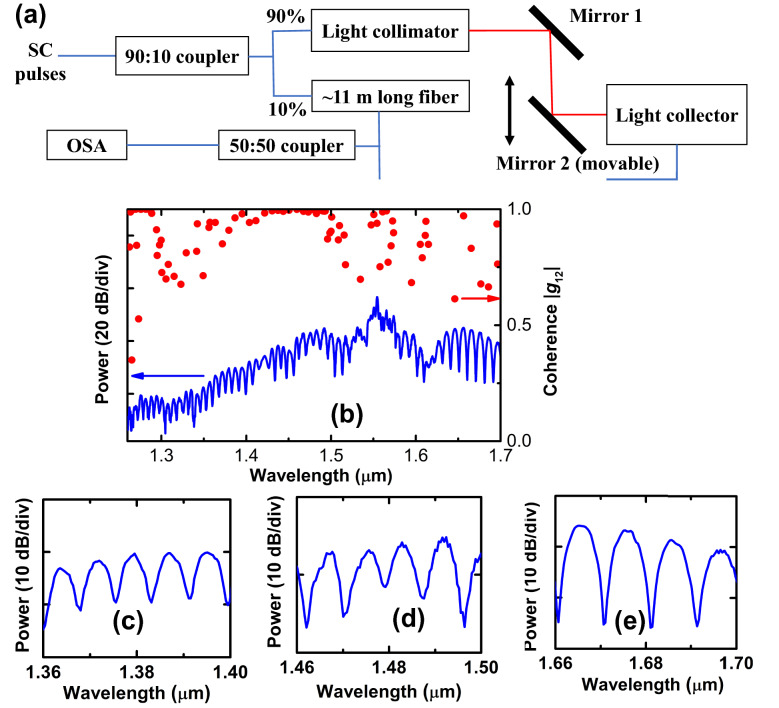
(**a**) Schematic of the experimental setup for the coherence measurements, where the blue lines donate PM fibers, and the red lines show the light propagating path in the air. (**b**) Experimentally measured spectral coherence (red circles) and fringes (blue line). Magnified fringe spectrum at 1.38 µm (**c**), 1.48 µm (**d**) and 1.68 µm (**e**), each with a 40 nm-span.

Next, we experimentally characterized the spectral coherence of the generated spectrum from the USRN waveguide using an asymmetric Michelson interferometer. The setup interferes two back to back SCG pulses. Figure 5a shows the schematic of the experimental setup for the coherence measurements, where the SC pulses generated in the USRN waveguide are firstly coupled into a 90:10 coupler and are split into two different arms. In one arm, 90% of the pulse power is coupled into a light collimator prior to Mirror 1, reflected by Mirror 1, travels to Mirror 2 and is then collected by a light collector. In this arm, Mirror 1 is position fixed, while Mirror 2 is fixed onto a motorized stage to provide the tunability of the time delay. In the other arm, 10% of the pulse power is coupled into a long fiber with a length of ~ 11 m, which is used to introduce an additional time delay. The two different pulses (back to back) in each arm are combined with a 50:50 coupler and interfere with each other. Finally, the output spectrum from the 50:50 coupler is measured using an optical spectrum analyzer (OSA).

Figure 5b shows the experimentally obtained spectral coherence (red circles) and fringes (blue line). The magnified fringe spectra at 1.38 µm, 1.48 µm, and 1.68 µm are respectively shown in Fig. 5c–e, each with a 40 nm-span. In Fig. 5b–e, we can see clear fringes with good fringe visibility that demonstrate the spectral coherence of the generated broadband spectrum. The degree of coherence can be quantified with a parameter |*g*_12_|, calculated using Eq. (3)

3 $$\left| {g_{12} (\lambda )} \right| = {{\left( {I_{1} (\lambda ) + I_{2} (\lambda )} \right)V(\lambda )} \mathord{\left/ {\vphantom {{\left( {I_{1} (\lambda ) + I_{2} (\lambda )} \right)V(\lambda )} {2\sqrt {I_{1} (\lambda )I_{2} (\lambda )} }}} \right. \kern-\nulldelimiterspace} {2\sqrt {I_{1} (\lambda )I_{2} (\lambda )} }}$$

In Eq. (3), *I*_1_(*λ*) and *I*_2_(*λ*) refer to the optical intensities of light propagating in the two different arms of the interferometer, while *V*(*λ*) denotes the fringe visibility calculated by *V*(*λ*) = (*I*_max_—*I*_min_)/ (*I*_max_ + *I*_min_)^11,13^. Due to the low pump power, the high coupling losses when coupling from free space to fiber, and the limited bandwidth of components used in the asymmetric Michelson interferometer, the wavelength range where the coherence fringes could be observed utilizing the OSA is limited to 1260 nm to 1700 nm, as shown in Fig. 5b. Similar limitations regarding coherence measurements have also been described in Ref. 13. We note that the spectral coherence |*g*_12_| of the SCG here is characterized to have an average value larger than 0.90 across the measured wavelength span from 1260 to 1700 nm.

## Discussion and conclusions

Table 1 shows recent demonstrations of SCG in various on-chip platforms. As shown in Table 1, a considerably wider pulse width of 500 fs is used in this work compared with other SCG demonstrations, allowing the pulse width requirements for the generation of supercontinuum to be relaxed. In addition, the pulse energy used in this work is only 17 pJ, considerably smaller than those reported in Lithium niobate (800 pJ)^12^, Si_3_N_4_ (25.5 pJ, 1.4 nJ, and 87.4 pJ)^14–16^ and silicon rich nitride (105 pJ)^17^. Although SCG demonstration in Si^7^ required smaller pulse energy (4.5 pJ), no coherence characterization was shown. Comparing the various platforms, the SCG demonstrated in AlGaAs waveguides^13^ required the lowest pulse energy of 3.6 pJ. AlGaAs while not CMOS-compatible, also possesses similar favorable nonlinear optical properties as USRN, namely a high Kerr nonlinearity and absence of TPA at telecommunications wavelengths^32^. In addition, unlike that reported in Si^7,9^ and Si_3_N_4_^14,16^ which utilized pump wavelengths of 2.5 µm, 1.9 µm, 1030 nm, and 795 nm, respectively, the pumping wavelength used in our USRN waveguide lies within telecommunications wavelengths where light sources are cheap and easily accessible.

**Table 1:** Comparison of recent experimental demonstrations of SCG in various on-chip platforms.

Platform	Device dimension	Pulse width	Pulse energy	SCG bandwidth	Coherence	References
Si	L = 20 mm 1210 × 320 nm	300 fs @2.5 um	4.5 pJ	1.3-octave@–20 dB (1510–3670 nm)	Not measured	7
Si	L (not mentioned) 920 × 315 nm	50 fs @1.9 um	18 pJ	1.1-octave@–20 dB (1124–2400 nm)	Average |*g*_12_|= ~ 0.90	9
Lithium niobate	L = 10 mm 1750 × 600 nm	200 fs @1560 nm	800 pJ	1.5-octave@–40 dB (700–2200 nm)	Not measured	12
AlGaAs	L = 3 mm 500 × 300 nm	100 fs @1555 nm	3.6 pJ	1-octave @–40 dB (1055–2155 nm)	Average |*g*_12_|= ~ 0.85	13
Si_3_N_4_	L = 8 mm 950 × 690 nm	92 fs @1030 nm	25.5 pJ	1.5-octave@–40 dB (673–1944 nm)	Average |*g*_12_|= ~ 0.96	14
Si_3_N_4_	L = 6 mm 1000 × 900 nm	120 fs @1560 nm	1.4 nJ	2.2-octave@–30 dB (526–2600 nm)	Not measured	15
Si_3_N_4_	L = 10 mm 500 × 300 nm	100 fs @795 nm	87.4 pJ	1-octave@ –30 dB (488–978 nm)	Not measured	16
Silicon rich nitride	L = 10 mm 1650 × 700 nm	130 fs @1555 nm	105 pJ	1.5-octave@–30 dB (820–2250 nm)	Not measured	17
USRN (Si_7_N_3_)	L = 3 mm 600 × 400 nm	500 fs @1555 nm	17 pJ	1006–2240 nm@–40 dB level, with components from 1072 to 1344 nm below the – 40 dB level	Average |*g*_12_|= ~ 0.90	This work

In conclusion, the SCG demonstrated in this work relaxes the pulse width requirement, requires low pulse energy, and shows good coherence, making it a favorable light source in various applications, including self-referencing, metrology, and imaging. Furthermore, due to its CMOS-compatibility, larger linear refractive index, and small device footprint, the USRN waveguide in this work may also be integrated with devices providing optical functions in other important domains, such as achromatic metadevices^33^, broadband, achromatic metalenses^34^ and high-sensitivity sensing^35^.

## Methods

In the spectral broadening experiment, a mode-locked femtosecond fiber laser is utilized to launch 500 fs pulses centred at 1555 nm, followed by an EDFA. The pulses are adjusted for quasi-TE polarization and coupled into the waveguide. The output spectra from the waveguide are separately monitored utilizing two different OSAs, one for the wavelength range from 800 to 1750 nm, and the other for the wavelength range from 1600 to 2400 nm.

In the spectral coherence characterization experiment, we used an asymmetric Michelson interferometer, where two different SCG pulses in time interfere with each other. The output spectrum of the Michelson interferometer is coupled into a fiber and measured by an OSA.

The USRN waveguide was implemented on a 10-μm SiO_2_ layer on a Si substrate. A 400 nm thick USRN film is deposited using inductively coupled chemical vapor deposition at a temperature of 250 °C. A channel waveguide structure is patterned using electron-beam lithography and transferred to the USRN layer via inductively coupled plasma etching. Finally, the deposition of a 2-µm SiO_2_ cladding was performed using plasma-enhanced chemical vapor deposition.
